# Morphological and functional parameters in X-linked retinoschisis patients–A multicentre retrospective cohort study

**DOI:** 10.3389/fmed.2023.1331889

**Published:** 2024-01-11

**Authors:** Peter Kiraly, Immanuel P. Seitz, Maram E. A. Abdalla Elsayed, Susan M. Downes, Chetan K. Patel, Peter Charbel Issa, Johannes Birtel, Luca Mautone, Simon Dulz, Yevgeniya Atiskova, Philipp Herrmann, Nika Vrabič, Martina Jarc-Vidmar, Marko Hawlina, M. Dominik Fischer

**Affiliations:** ^1^Oxford Eye Hospital, Oxford University Hospitals NHS Foundation Trust, Oxford, United Kingdom; ^2^Nuffield Laboratory of Ophthalmology, University of Oxford, Oxford, United Kingdom; ^3^Centre for Ophthalmology, University Hospital Tübingen, Tübingen, Germany; ^4^Department of Ophthalmology, University Medical Center Hamburg-Eppendorf, Hamburg, Germany; ^5^Department of Ophthalmology, University Hospital Bonn, Bonn, Germany; ^6^Eye Hospital, University Medical Centre Ljubljana, Ljubljana, Slovenia

**Keywords:** X-linked retinoschisis, XLRS, morphological parameters, functional parameters, symmetry between eyes, carbonic anhydrase inhibitors, gene therapy, trial endpoints

## Abstract

**Introduction:**

X-linked retinoschisis (XLRS) is a potential target for gene supplementation approaches. To establish potential structural and functional endpoints for clinical trials, a comprehensive understanding of the inter-eye symmetry, relationship between structural and functional parameters, and disease progression is vital.

**Methods:**

In this retrospective multicentre study, 118 eyes of 59 XLRS patients with *RS1* mutations were assessed. Information from center databases included: *RS1* variant; age at presentation; best-corrected visual acuity (BCVA), central retinal thickness (CRT), macular volume (MV) at presentation and at the last follow up; full-field electroretinogram (ERG) findings; presence of peripheral retinoschisis and complications (vitreous hemorrhage, retinal detachment); treatment with systemic or topical carbonic anhydrase inhibitors (CAI).

**Results:**

Inter-eye symmetry revealed strong correlation in CRT (*r* = 0.77; *p* < 0.0001) and moderate correlations in MV (*r* = 0.51, *p* < 0.0001) and BCVA (*r* = 0.49; *p* < 0.0001). Weak or no correlations were observed between BCVA and structural parameters (CRT, MV). Peripheral retinoschisis was observed in 40 (68%), retinal detachment in 9 (15%), and vitreous hemorrhage in 5 (8%) patients, respectively. Longitudinal examinations (mean, 4.3 years) showed no BCVA changes; however, a reduction of the CRT (*p* = 0.02), and MV (*p* = 0.01) was observed. Oral and/or topical CAI treatment did not significantly alter the CRT (*p* = 0.34).

**Discussion:**

The XLRS phenotype demonstrates a strong CRT symmetry between the eyes within individual patients and stable BCVA over several years. BCVA exhibits a weak correlation with the morphological parameters of retinal thickness (CRT MV). In our cohort, longitudinal functional changes were not significant, likely attributed to the short average follow-up period. Furthermore, CAI treatment didn’t influence both morphological and functional outcomes.

## 1 Introduction

X-linked retinoschisis (XLRS) is a monogenic inherited retinal disease (IRD) caused by mutations in the *RS1* gene and has a prevalence of 1/5,000 to 1/20,000 in males. XLRS is fully penetrant and associated with variable phenotypes (1). Characteristic findings include symmetric disease presentation (96%), macular schisis (82%), macular atrophy (11%), peripheral retinoschisis (39%), and vitreous hemorrhages and/or retinal detachment (19%) (2). On optical coherence tomography (OCT), intraretinal cystoid cavities (ICC), usually located in the fovea and within the inner nuclear layer, are commonly observed (2, 3). Moreover, changes in the photoreceptor outer segment length, disruption of external limiting membrane (ELM) and ellipsoid zone (EZ) may be observed (4, 5). Lin et al. showed significant correlations between the best corrected visual acuity (BVCA) and central retinal thickness (CRT; 1 mm) but not between BCVA and macular volume (MV; 6 mm) (6). Thinner photoreceptor outer segment length, disruption of EZ and ELM have been associated with worse BCVA (5, 6). On full-field electroretinography (ERG), XLRS commonly presents with an abnormal b-to-a wave amplitude ratio and a fully electronegative pattern (5).

XLRS causes progressive vision loss, which often becomes apparent in childhood and may lead to severely impaired visual function and blindness (5). Early onset XLRS is associated with severe phenotype and poor visual function (7). The largest retrospective observational study showed that in 50% of patients low vision (20/70 > visual acuity (VA) ≥20/200) developed by the age of 25. Severe visual impairment (20/200 > VA ≥ 20/400) and blindness (VA < 20/400) developed in approximately 20% of patients throughout their lifetime (5).

As of now, there are no large prospective studies reporting an effective treatment for patients with XLRS. Systemic and topical carbonic anhydrase inhibitors (CAI) have shown to reduce the volume of ICC in some patients, however, this has not been proven on larger cohorts of XLRS patients (4). Regarding surgical approaches, a study indicated that vitrectomy can improve BCVA, reduce macular schisis, and help prevent complications (8). Two intravitreal human adeno-associated virus (AAV) based gene therapy trials had an acceptable safety profile, but lacked convincing morphological or functional improvement (9, 10). With several gene therapy trials emerging for IRD (11), it is important to understand the natural course of IRD to establish optimal therapeutic window and realistic functional and morphological endpoints. With prospective studies lacking, a better understanding of the natural course of XLRS using large cohorts would provide meaningful insights.

In this study, we evaluate functional and morphological symmetry between both eyes in XLRS patients to determine if the non-treated eye could serve as an internal control in an interventional trial. Moreover, we wanted to determine associations between morphological and functional parameters and quantify longitudinal changes of putative endpoints in the absence of treatment and in case of current best management. We applied a subgroup analysis to determine morphological and functional parameters across different age groups. To evaluate usefulness of CAI treatment, we compared morphological and functional changes in treated vs. untreated eyes.

## 2 Materials and methods

The retrospective data was obtained between 2004 and 2023 from the Oxford Eye Hospital; the Department of Ophthalmology, University Medical Center Hamburg-Eppendorf; the Department of Ophthalmology, University Hospital Bonn; the University Eye Hospital Ljubljana. The diagnosis of XLRS was established by an IRD specialist (12) through an evaluation of medical records, slit lamp biomicroscopy, multimodal imaging and full-filed ERG. All patients underwent genetic testing which confirmed a pathogenic *RS1* variant (13). A systematic review of databases was performed with the collection of: variant of *RS1* mutation; patients’ age at diagnosis; BCVA, CRT, MV at presentation and at the last follow up; full-field ERG findings; presence of peripheral retinoschisis and complications (vitreous hemorrhage, retinal detachment); potential treatment with systemic or topical CAI; potential surgical intervention.

BCVA was assessed using Snellen charts, and the recorded values were subsequently converted into logMAR units to facilitate statistical analysis. In 50 patients, OCT scans were performed with the Spectralis imaging platform (Spectralis, Heidelberg Engineering, Heidelberg, Germany), while Topcon imaging platform (3D OCT-1000; Topcon Medical Systems, Japan) was used in 9 patients. When advanced retinal pathology led to inaccurate automatic segmentation, a medical retina specialist conducted manual segmentation. After segmentation correction, the CRT (1 mm) was measured. MV was defined as the sum of all subfield volumes within the 6 mm diameter of the ETDRS circle (14).

Full-field ERG measurements were in accordance with the standards outlined by the International Society for Clinical Electrophysiology of Vision (ISCEV) and categorized as either normal, significantly reduced b-to-a wave amplitude ratio, or electronegative (15).

Statistical analyses were performed using SPSS Statistics for Windows, version 28 (IBM, Armonk, NY, USA), employing a significance threshold of *p* < 0.05 to determine statistical significance. The Pearson correlation coefficient was used to assess the correlations between both eyes, as well as between morphological and functional parameters within the same eye. Linear mixed-effects models (LMEM), fit by restricted maximum likelihood estimates (REML) were used to assess the possible effect of the predictor variables age at examination ([y], pharmacologic treatment [yes; no], and the time since baseline [years], as well as plausible effect interactions [e.g., pharmacological treatment*age at examination], on the variance of the dependent variables BCVA, CRT and MV. To account for repeated measurements, the use of data from two eyes of the same individual, and inter-individual variability, the patient ID and eye laterality (nested into patient ID) were set as random effects. The LMEMs were described by the following formula:


$$Y_{ijk}=\text{μ + }age\text{ + }treatment_{i}\;+timesince\;BL_{j\left(i\right)}+eye_{k\left(i,j\right)}{\text{ + ε}}_{ijk}$$

The LMEMs used for analysis were visually inspected for violation of homoscedasticity, and no gross violations were detected. In statistical testing, the Brown-Forcythe test revealed a minor violation for the BCVA model only (*p* = 0.0102). For CRT and MV there was no violation homoscedasticity. As LMEMs are known to be relatively robust against violations of distributional assumptions, and the BCVA model was in line with a purely descriptive analysis, the BCVA model was not discarded. Overall, the largest share of the variances in BCVA, CRT and MV could be attributed to test–retest variability and the random effects patient ID and laterality (BCVA: 75.3%, CRT: 82.9%, MV: 90.7%).

## 3 Results

### 3.1 Clinical findings and genetic characteristics

The study included 118 eyes from 59 XLRS patients, who were, on average, 24 years old at presentation, with ages ranging from 1 to 73 years. Follow-up data were available for 44 patients, with an average duration of 4.25 years. Peripheral retinoschisis was observed in 40 (68%), retinal detachment in 9 (15%), and vitreous hemorrhage occurred in 5 (8%) patients. Average BCVA (logMAR) at presentation was 0.60 (OD, right eye), and 0.62 (OS, left eye), respectively. Average CRT and MV at presentation were 402 μm and 8.8 mm^3^ (OD), and 409 μm and 9.0 mm^3^ (OS), respectively. Among the treatment approaches, 13 patients (22%) received topical CAI, 4 patients (7%) were treated with systemic CAI, and 6 patients (10%) received a combination of topical and systemic CAI. Furthermore, 13 patients (22%) had a history of vitreoretinal procedures related to XLRS complications (vitreous hemorrhage, retinal detachment). On full-field ERG (*n* = 20), 17 patients had an electronegative ERG, 3 patients had a reduced b-to-a wave amplitude ratio. In electronegative ERG, a reduced photopic and scotopic b-wave amplitude was observed, reflecting functional impairment in the inner retinal layers. The a-wave (although not infrequently subnormal in amplitude) was considerably less impaired than b wave, creating a negative ERG pattern. In later stages, marked impairment of both a- and b-wave ERG amplitudes were found. Genetic analysis revealed 39 different variants in the *RS1* gene. The most frequently identified variant (*n* = 6) was c.214G > A p.(Glu72Lys).

### 3.2 Symmetry of disease between eyes

**FIGURE 1:**
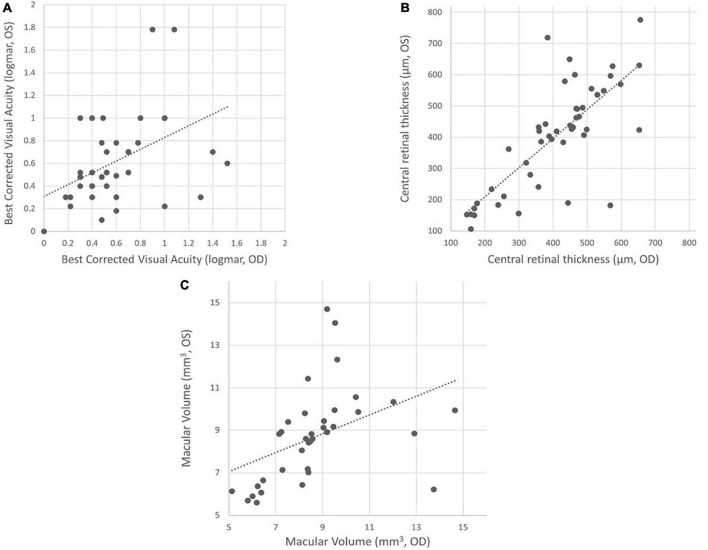
Functional and morphological symmetry between both eyes in XLRS patients. **(A)** Best corrected visual acuity (BCVA) (*r* = 0.49; *p* < 0.01), **(B)** central retinal thickness (CRT) (*r* = 0.77; *p* < 0.01), and **(C)** macular volume (MV) (*r* = 0.51; *p* < 0.01), indicating a moderate to strong association between both eyes in the same patient.

Intraindividual symmetry between eyes was assessed for BCVA, CRT, and MV. All these parameters exhibited a highly significant correlation (*p* < 0.0001) between the right and left eye. The Pearson’s correlation coefficients (*r*) ranged from 0.5 to 0.8, indicating a moderate to strong association (Figure 1). These findings remained consistent over time, with similar results observed at both the baseline and last visit. Consistently, there were highly significant correlations (all *p* < 0.0001) and strong associations [range: 0.63–0.87 (*r*)] observed between BCVA, CRT, and MV measurements from the baseline to the last visit (Table 1).

**TABLE 1:** Intraindividual symmetry, and stability of findings over time [between baseline (BL) and last visit (LV)].

Symmetry	*r* (Pearson)	*N*	Lower 95%	Upper 95%	*p*-value
BCVA	0.49	89	0.31	0.63	<0.01
CRT	0.77	87	0.67	0.84	<0.01
MV	0.51	66	0.31	0.67	<0.01
**BL vs. LV**	***r* (Pearson)**	***N***	**Lower 95%**	**Upper 95%**	***p*-value**
BCVA	0.72	75	0.59	0.81	<0.01
CRT	0.87	71	0.80	0.91	<0.01
MV	0.63	50	0.43	0.77	<0.01

There was a strong correlation in best corrected visual acuity (BCVA); central retinal thickness (CRT); macular volume (MV) between eyes of the same individual, and between an individual’s first and last visit. N = sample size, lower/upper 95% confidence interval boundaries, p-value (pairwise significance).

### 3.3 Correlations between morphological and functional parameters

**FIGURE 2:**
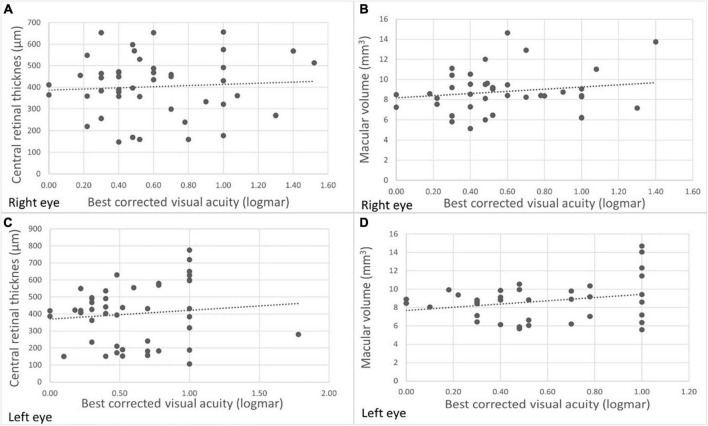
Functional and morphological correlations in XLRS patients at baseline for the right and left eyes. There were no significant correlations between best corrected visual acuity (BCVA) and central retinal thickness (CRT) in both the right eye **(A)** (*r* = 0.06; *p* = 0.66) and the left eye (*r* = 0.16; *p* = 0.30) **(C)**. Additionally, no significant correlation was observed between BCVA and macular volume (MV) in the right eye (*r* = 0.23; *p* = 0.11) **(B)**. However, a statistically significant correlation was evident between BCVA and MV in the left eye **(D)** (*r* = 0.42; *p* < 0.01).

BCVA did not correlate with CRT (OD: *p* = 0.66; OS: *p* = 0.30) (Figures 2A, C). Additionally, there were no correlations observed between MV and BCVA in the right eye (*p* = 0.11) (Figure 2B). A significant positive correlation was noted between MV and BCVA in the left eye (*p* < 0.01) (Figure 2D).

### 3.4 Morphological and functional parameters in different age group cohorts

**FIGURE 3:**
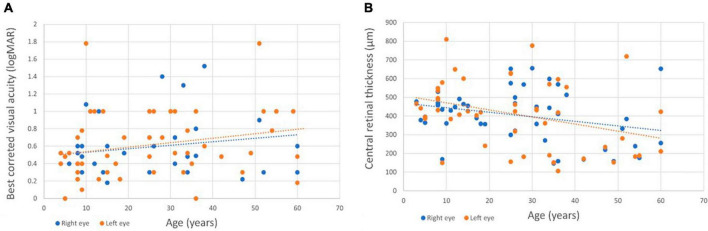
Changes in best corrected visual acuity (BCVA) and central retinal thickness (CRT) across age. **(A)** BCVA changes with age (*r* = 0.20, *p* = 0.15 in the right eye; *r* = 0.23, *p* = 0.09 in the left eye). **(B)** CRT changes with age (*r* = –0.29, *p* = 0.04 in the right eye; *r* = –0.35, *p* = 0.01 in the left eye).

**FIGURE 4:**
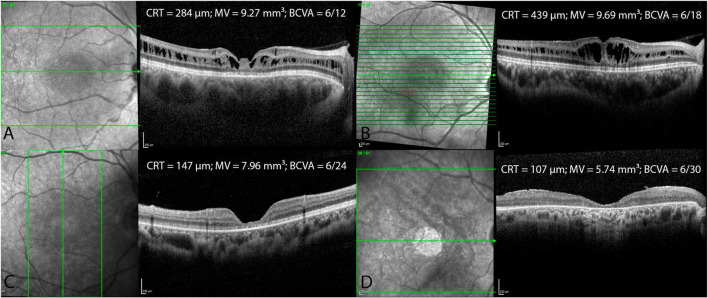
Foveal optical coherence tomography (OCT) scans in XLRS patients with central retinal thickness (CRT), macular volume (MV) and best corrected visual acuity (BCVA) measurements in four different patients **(A–D)**.

Table 2 displays morphological and functional parameters in XLRS patients, grouped by age at baseline. In the pediatric/adolescent group (0–18 years), the retina was notably thicker compared to normative values (16), with the best BCVA among the three age group cohorts. Patients in the middle-age group (18–40 years) exhibited retinal thickness similar to the pediatric/adolescent group; however, BCVA was lower in both eyes when compared with the pediatric/adolescent group. The oldest age group cohort (40–73 years) displayed the worst BCVA and the thinnest retina. Figure 3 shows BCVA and CRT changes across age. Figure 4 presents foveal OCT scans in XLRS patients alongside corresponding retinal thickness measurements (CRT, MV) and BCVA. The first XLRS patient (Figure 4A) displayed moderate volume of ICC with a very good BCVA. As the volume of ICC increased (Figure 4B), there was a corresponding deterioration in BCVA. As the ICC collapsed (Figure 4C), retinal thickness decreased, which was associated with a loss of integrity in the outer retinal layers and additional vision loss. An older patient (Figure 4D) exhibited an atrophic retina without ICC and demonstrated poor BCVA.

**TABLE 2:** Morphological and functional parameters in XLRS patients, grouped by their age at baseline.

Age group cohort	0–18 years	18–40 years	40–73 years
Number of patients	29	19	11
x̄ (SD), years	8.2 (4.8)	30.2 (5.2)	54.7 (8.3)
RE BCVA x̄ (SD), log mar	0.50 (0.27)	0.72 (0.14)	0.83 (0.68)
LE BCVA x̄ (SD), log mar	0.52 (0.39)	0.67 (0.30)	0.96 (0.81)
RE CRT x̄ (SD), μm	427 (83)	438 (152)	281 (149)
LE CRT x̄ (SD), μm	476 (131)	402 (194)	285 (182)
RE MV x̄ (SD), mm^3^	8.76 (1.42)	9.26 (2.40)	7.94 (2.70)
LE MV x̄ (SD), mm^3^	9.54 (3.31)	9.11 (2.24)	7.83 (3.07)

right eye (RE); left eye (LE); best corrected visual acuity (BCVA); central retinal thickness (CRT); macular volume (MV); mean (x̄); standard deviation (SD).

### 3.5 Estimation of progression rates, and effects of pharmacological treatment, age at baseline, and time since baseline on BCVA, CRT, and MV

#### 3.5.1 Best corrected visual acuity

There were no significant effects on visual acuity. Our model estimated a change of BCVA of + 0.0001/year (LogMAR). Using linear regression, the rate of change was estimated at + 0.003/year. In summary, our sample of patients did not exhibit a change in BCVA over time.

#### 3.5.2 Central retinal thickness

**FIGURE 5:**
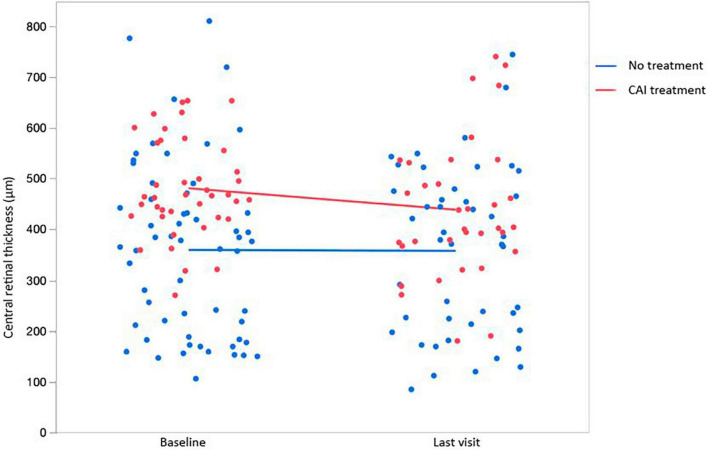
Changes in central retinal thickness (CRT) from baseline (BL) to the last visit (LV) in association with the use of topical and/or systemic carbonic anhydrase inhibitors (CAI).

**FIGURE 6:**
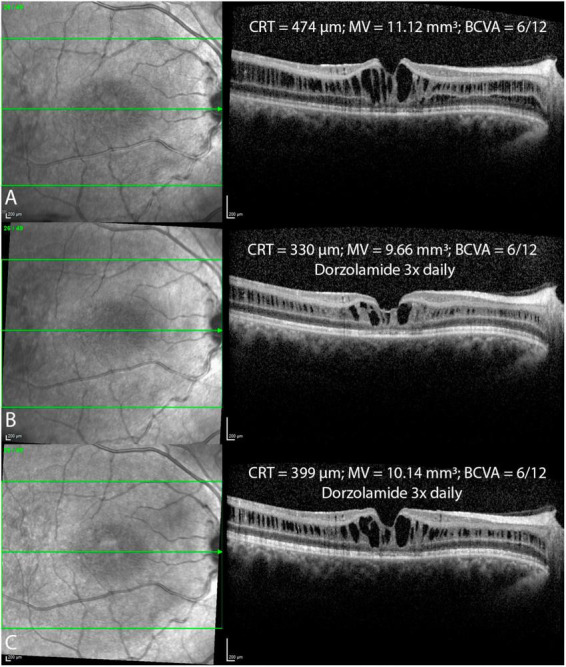
Foveal optical coherence tomography (OCT) scans in a patient before **(A)** and after the topical treatment with dorzolamide drops 3x daily **(B,C)** over a one year follow up period reveal fluctuations in intraretinal cystoid cavities, with ongoing treatment. Central retinal thickness (CRT); macular volume (MV); best corrected visual acuity (BCVA).

In our cohort of patients, several tested variables were associated with statistically significant CRT changes. Age at baseline: For every year in the age of patients at their baseline visit, CRT was 2.80 μm thinner (*p* = 0.0077). Time since baseline: The estimated rate of progression was −4.94 μm/year (*p* = 0.0156). Pharmacologic treatment: Treated retinas were overall 41.2 μm thicker, compared to untreated retinas (*p* = 0.0257). As expected, laterality was not a significant factor (−5.88 μm in right eyes, *p* = 0.4478). In addition, pharmacological treatment did not significantly influence the yearly change of CRT (Δ + 1.92 μm/year in untreated eyes compared to treated eyes, *p* = 0.3394) (Figure 5). Fluctuations in ICC were observed in patients under continuous treatment with CAI (Figure 6).

#### 3.5.3 Macular volume

For MV, only time since baseline was a significant effect. Estimated rate of progression was −0.076 mm^3^/year (*p* = 0.0138).

## 4 Discussion

In this study, a moderate to strong correlations in symmetry between eyes in BCVA, CRT, and MV is described in XLRS patients. Conversely, weak or no correlations were observed between baseline functional (BCVA) and morphological (CRT, MV) parameters. In our subgroup analysis, morphological and functional disparities across different age groups were revealed. Longitudinal analysis showed no significant BCVA changes over time. However, there was a significant reduction in retinal thickness (CRT, MV). Furthermore, the pharmacological treatment did not have a significant impact on the annual change in CRT when comparing untreated eyes to treated eyes.

### 4.1 Evaluation of symmetry between eyes

High inter-eye symmetry was observed in several IRDs (17–20), which may allow the utilization of the fellow eye as a control in interventional studies. Here, CRT displayed a strong association (*r* = 0.77, *p* < 0.01), whereas BCVA showed a moderate association (*r* = 0.49, *p* < 0.01) between the measurements in the respective eyes. These findings align with the results of large retrospective studies involving patients with XLRS (2, 5, 21). Only one study conducted by Fenner et al. reported a lack of significant correlation between the two eyes in BCVA (*r* = 0.17, *p* = 0.08), and a relatively weak correlation in CRT (*r* = 0.38, *p* < 0.01) (22). Hence, if the contralateral eye is employed as a control in an interventional trial, CRT could be a more suitable parameter compared to BCVA due to its notably lower inter-eye variability. Considering the dynamic characteristics of morphological and functional parameters in XLRS patients, inter-eye symmetry could potentially change throughout the follow-up period. Nonetheless, our results regarding symmetry remained consistent over time, with comparable correlations observed both at the baseline and during the final visit.

### 4.2 Clinical characteristics of the study cohort

Our cohort of patients included a wide age range (1–73 years) at presentation, thus encompassing various phenotypes and functional outcomes that develop throughout the natural course of XLRS. However, the average age at presentation in our patient group was 24 years, indicating that the majority of our XLRS patients exhibited a pediatric/young adult phenotype. Our patients exhibited low vision at the time of presentation, as per the World Health Organization classification (5). CRT was above the normative values, and MV was at the higher end of normal range when compared to normal controls (14). In our cohort, peripheral retinoschisis was observed more frequently (68%) compared to the rates reported in previous large retrospective studies, which ranged from 38.9 to 51.9% (2, 5, 22). The rate of vision-threatening complications, such as retinal detachment and vitreous hemorrhage, in our cohort closely aligned with the findings reported in previous studies (1, 2, 5, 23).

### 4.3 Morphological and functional parameters in three age group cohorts

In our study, we analyzed morphological and functional parameters in XLRS patients across three age group cohorts (pediatric, middle-age, and older) to better understand the natural course. In pediatric patients, there was a notable thickening of the retina along with mild visual impairment. Within the middle-age cohort, the retinal thickening persisted while BCVA deteriorated to a moderate level of impairment. In the oldest group, the ICC collapsed, leading to concurrent retinal thinning and atrophy, which further exacerbated the decline in BCVA.

In patients with XLRS, the loss of *RS1* function leads to splitting within the retinal layers and ICC formation, disrupting visual signal processing and contributing to progressive vision loss (24, 25). ICC within the inner nuclear layer were observed using handheld spectral domain OCT in 15 out of 16 eyes in very young patients (aged 7 months to 10 years) (26). Hence, due to the early macular morphological involvement (26, 27), BCVA is markedly reduced in pediatric patients at presentation, as indicated by prior publications (5, 22) and confirmed by our findings. The largest retrospective study demonstrated that BCVA remains stable until the age of 20, followed by a gradual decline thereafter, with approximately half of the patients developing low vision by the age of 25 (5). Our findings in the XLRS middle-age groups revealed a reduction in BCVA compared to the pediatric group, alongside relatively minor changes in MV and CRT. We hypothesize that persistent ICC contribute to compromised retinal signal transmission and/or development of outer retinal alterations, including photoreceptor outer segment length thinning, disruption of ELM, EZ, or diffuse outer retinal atrophy, all of which are recognized to correlate with poorer BCVA (5, 6, 22, 28, 29). It remains uncertain whether outer retinal changes arise as a consequence of ICC or progress independently from them. Furthermore, it remains unclear whether the volume of ICC influences the extent of outer retinal changes. In older patients, the collapse of ICC and the outer retinal atrophy development are observed (2, 5, 22) (Figure 4D), leading to retinal thinning and an additional deterioration in BCVA as shown in our older XLRS cohort. Therefore, when patients present at an older age, XLRS can mimic geographic atrophy in dry age-related macular degeneration, much like certain other macular dystrophies (30).

### 4.4 Longitudinal changes in morphological and functional parameters

Regarding the longitudinal data, BCVA remained unchanged in our cohort throughout the 4.25-year follow-up period. This finding is consistent with previous studies and a median follow-up from 5.7 to 12 years (2, 22, 31). Among the published studies, only Hahn et al. observed a significant annual decline in visual acuity over a mean follow-up period of 13.2 years (5). Regarding longitudinal morphological parameters in our cohort, significant retinal thinning was observed as evidenced by a significant reduction in CRT and MV that is most likely attributed to the collapse of ICC and the development of retinal atrophy.

### 4.5 Correlations between morphological and functional parameters

In our study, a very weak correlation was observed between CRT and BCVA, while a weak to moderate correlation was found between MV and BCVA. Our study aligns with Fenner et al., who did not find significant correlations between CRT and BCVA (22). However, two other studies did establish a notable correlation between CRT and BCVA but not between MV and BCVA (6, 29). CRT and MV are in XLRS patients influenced by volume of ICC and atrophy of the outer retinal layers. As the volume of ICC increases, both CRT and MV show an increase, while BCVA decreases (Figures 4A, B). Conversely, with the progression of outer retinal atrophy (Figures 4C, D), there is a decrease observed in CRT, MV, and BCVA. Therefore, while CRT and MV measurements are easily obtained, they do not accurately represent BCVA. On the other hand, parameters that reflect the integrity of the outer retinal layers (such as photoreceptor outer segment length, ELM, EZ) are not standardized and need to be measured manually. Nevertheless, these parameters show a much stronger correlation with BCVA (2, 5, 6, 22, 28, 29).

### 4.6 Effects of pharmacological treatment

In our study, pharmacological treatment with CAI did not significantly affect CRT, MV, or BCVA. However, our study had several limitations due to its retrospective nature, including the utilization of various treatment options (topical; oral; topical and oral) as well as variations in the duration of treatment. The proposed mechanism of CAI therapy involves facilitating the movement of fluids out of the retina, leading to a reduction in the size of cystic cavities and potentially promoting cellular adhesion (32). The only prospective study by Pennesi et al. exhibited no morphological or functional improvement over an 18-month follow-up in XLRS patients undergoing CAI therapy (33). On the other hand, several retrospective studies have reported a reduction in ICC volume along with improvement in BCVA in a few (22, 32, 34–37). However, these studies employed varied treatment protocols and follow-up durations with no control group. ICC is prone to substantial spontaneous fluctuations, as evidenced by Mautone et al., who observed significant diurnal changes in macular thickness and visual function (38). Campbell et al. showed complete spontaneous resolution of ICC with significant vision improvement in two case reports (6 years; 23 years). Fluctuations were observed in one of our patients (Figure 6) as well, where 6 months after treatment with dorzolamide eye drops (3x daily), a notable reduction in ICC was evident (Figure 6B). However, in the subsequent follow-up 6 months later, despite the patient’s continued use of the same treatment, there was an increase in the volume of ICC (Figure 6C). In our study, CRT in treated patients was statistically significantly higher than in untreated patients. This could be associated with the observation that patients with thicker retinas, who are more likely to receive treatment, have a higher volume of ICC. In contrast, thinner retinas tend to have an insignificant ICC volume and are typically managed with observation alone. Moreover, these observations might indicate a treatment efficacy bias, where treatment is initiated upon an increase in ICC, anticipating a reduction of these cavities at follow-up, even without CAI treatment. In absence of a randomized controlled trial, it is difficult to discern treatment effect from a simple regression to the mean (39).

#### 4.6.1 Morphological, functional endpoints, and gene therapy for XLRS update

Natural disease history studies are crucial for defining endpoints in clinical trials. In XLRS, no significant progression in retinal structure and function was observed from childhood to adulthood with a mean follow-up of 12 years (31). Hence, this period could represent an optimal window for gene therapy intervention to minimize the impact of natural course changes. Regarding functional endpoints, BCVA might not be suitable due to poor symmetry between both eyes, the very slow rate of deterioration, and the lack of improvement after CAI treatment. On the other hand, retinal sensitivity as measured by microperimetry, has shown improvement following the reduction of ICC in XLRS patients (38), mirroring a pattern established with the resolution of intraretinal fluid in diabetic macular edema (40). Therefore, considering its significance as a crucial functional parameter in other gene therapy trials (41), microperimetry measurement could be considered the optimal choice for evaluating functional outcomes in a gene therapy trial for XLRS. In animal models, gene therapy for XLRS led to a sustained rescue of retinal morphology and the quick resolution of ICC (31, 42–44). A gene therapy study in *RS1* knockout mice showed long-term rescue of retinal morphology with intraretinal cystoid changes resolution (45). Long-term rescue of retinal morphology and function by AAV-Rs1h gene transfer may provide a basis for considering intervention in the homologous human XLRS condition. Therefore, in a potentially effective gene therapy for XLRS in humans, one might expect the short-term resolution of ICC and the long-term preservation of outer retinal layers compared to the contralateral eye. The volume of ICC is not accurately represented by CRT and MV measurements. Hence, means to accurately quantify ICC volume in XLRS such as AI driven tools should be considered, akin to their use in measuring intraretinal fluid in diabetic macular edema and wet age-related macular degeneration (46).

Two clinical trials using AAV-based gene augmentation for XLRS demonstrated no significant improvement in morphological or functional parameters (9, 10). Although the safety profile was generally favorable, the majority of patients developed mild to moderate intraocular inflammation, and three patients developed chronic uveitis (9). A new phase 1/2 gene therapy trial involving subretinal application of AAV-SPR for XLRS has been initiated by Atsena Therapeutics. Compared to intravitreal administration, subretinal application offers an advantage due to the immune-privileged nature of the subretinal space, the lower required dose and more favorable biodistribution (47). Subretinal delivery of AAV leads to better gene transduction occurring at the level of photoreceptors, Muller cells, and RPE cells (48). In *RS1* knockout mice, subretinal delivery of AAV2/4-*RS1* was found to be superior to the intravitreal approach (43). On the other hand, subretinal application is associated with more surgical complications including chorioretinal atrophy (49, 50). Subretinal application of conventional AAV might pose even greater risks in XLRS patients, given the pre-existing schitic macula and the relatively good vision often observed in younger patients. However, novel vector systems that spread laterally beyond the margins of the raised bleb may be applied outside the most fragile macular area while still providing the putative therapeutic effect of gene augmentation where it is needed (44).

In conclusion, our study showed strong correlation in CRT and moderate correlations in MV and BCVA between eyes. Weak or no correlations were observed between baseline BCVA and morphological parameters (CRT, MV). Our subgroup analysis revealed morphological and functional differences across three age-dependent XLRS cohorts. CAI treatment did not significantly affect CRT change when comparing treated and untreated eyes.

## Data availability statement

The original contributions presented in the study are included in the article/supplementary material, further inquiries can be directed to the corresponding author.

## Ethics statement

The studies involving humans were approved by the Research Ethics Committee (reference 08/H0302/96) in Oxford; the Ethik-Kommission of the Ärztekammer Hamburg and the Ethik-kommission of the Medizinische Fakultät der Rheinischen Friedrich-Wilhelms-Universität in Bonn; and the National Medical Ethics Committee of the Republic of Slovenia (protocol ID number: 0120-534/2021/3) in Ljubljana. The studies were conducted in accordance with the local legislation and institutional requirements. Written informed consent for participation in this study was provided by the participants’ legal guardians/next of kin. Written informed consent was obtained from the individual(s) for the publication of any potentially identifiable images or data included in this article.

## Author contributions

PK: Conceptualization, Data curation, Investigation, Methodology, Project administration, Visualization, Writing – original draft, Writing – review and editing. IS: Formal analysis, Methodology, Writing – review and editing. MA: Writing – review and editing. SuD: Writing – review and editing. CP: Writing – review and editing. PC: Writing – review and editing. JB: Writing – review and editing. LM: Writing – review and editing. SiD: Writing – review and editing. YA: Writing – review and editing. PH: Writing – review and editing. NV: Writing – review and editing. MJ-V: Writing – review and editing. MH: Writing – review and editing. MF: Conceptualization, Funding acquisition, Methodology, Resources, Supervision, Writing – review and editing.
